# Glucose-induced electrical activities and insulin secretion in pancreatic islet β-cells are modulated by CFTR

**DOI:** 10.1038/ncomms5420

**Published:** 2014-07-15

**Authors:** Jing Hui Guo, Hui Chen, Ye Chun Ruan, Xue Lian Zhang, Xiao Hu Zhang, Kin Lam Fok, Lai Ling Tsang, Mei Kuen Yu, Wen Qing Huang, Xiao Sun, Yiu Wa Chung, Xiaohua Jiang, Yoshiro Sohma, Hsiao Chang Chan

**Affiliations:** 1 Epithelial Cell Biology Research Center, Key Laboratory of Regenerative Medicine of Ministry of Education of China, CUHK—SJTU Joint Center for Human Reproduction and Related Disease, Faculty of Medicine, School of Biomedical Sciences, The Chinese University of Hong Kong, Hong Kong, China; 2 Sichuan University-The Chinese University of Hong Kong Joint Laboratory for Reproductive Medicine, Key Laboratory of Obstetric, Gynecologic and Pediatric Diseases and Birth Defects of Ministry of Education of China, West China Second University Hospital, Sichuan University, Chengdu 610041, China; 3 Lui Che Woo Institute of Innovative Medicine, Faculty of Medicine, The Chinese University of Hong Kong, Hong Kong, China; 4 Department of Endocrinology, Beijing Tongren Hospital, Capital Medical University, Beijing 100730, China; 5 Department of Pharmacology, Keio University School of Medicine, Shinjuku, Tokyo 160-8582, Japan

## Abstract

The cause of insulin insufficiency remains unknown in many diabetic cases. Up to 50% adult patients with cystic fibrosis (CF), a disease caused by mutations in the gene encoding the CF transmembrane conductance regulator (CFTR), develop CF-related diabetes (CFRD) with most patients exhibiting insulin insufficiency. Here we show that CFTR is a regulator of glucose-dependent electrical acitivities and insulin secretion in β-cells. We demonstrate that glucose elicited whole-cell currents, membrane depolarization, electrical bursts or action potentials, Ca^2+^ oscillations and insulin secretion are abolished or reduced by inhibitors or knockdown of CFTR in primary mouse β-cells or RINm5F β-cell line, or significantly attenuated in CFTR mutant (DF508) mice compared with wild-type mice. VX-809, a newly discovered corrector of DF508 mutation, successfully rescues the defects in DF508 β-cells. Our results reveal a role of CFTR in glucose-induced electrical activities and insulin secretion in β-cells, shed light on the pathogenesis of CFRD and possibly other idiopathic diabetes, and present a potential treatment strategy.

## Abstract (web summary)

Patients with cystic fibrosis harbour mutations in the CFTR chloride channel and often develop diabetes for reasons that are poorly understood. Here Guo *et al.* show that CFTR is involved in the regulation of glucose-stimulated insulin secretion from pancreatic β-cells.

## Graphical abstract

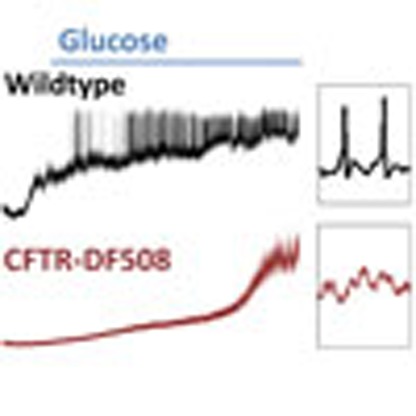

Diabetes mellitus or diabetes is a chronic metabolic disease with over 300 million people suffering worldwide, which could be either insulin insufficient (type 1 diabetes) or insulin resistant (type 2)^1^. Insulin insufficiency and impairment in pancreatic islet β-cells is also found combined with insulin resistance in type 2 diabetes^2^, the most prevalent form of diabetes. Although the cause of insulin insufficiency is generally considered to be a result of β-cell damage by autoimmunity, a high percentage of diabetic patients with insulin insufficiency show negative of those autoantibodies^3^. Notably, whereas most CFRD cases exhibit insulin insufficiency^4^,^5^, the exact cause remains elusive although destruction of the insulin-secreting pancreatic islets secondary to the obstruction of the pancreatic duct due to defective CFTR has long been considered the underlying cause^6^,^7^. Interestingly, CFTR expression in the pancreatic islet has been reported^8^; however, its exact role in islet function remains unexplored.

It is well known that insulin is secreted by the β-cells upon the elevation of blood glucose level. Glucose-stimulated insulin secretion is associated with a complex electrical activity in the pancreatic islet β-cell, which is characterized by a slow membrane depolarization superimposed with bursts of action potentials^9^. Closing adenosine triphosphate (ATP)-sensitive K^+^ channels (K_ATP_) in response to glucose increase is generally considered the initial event that depolarizes the β-cell membrane and activates the voltage-dependent Ca^2+^ channels^10^, leading to the increase in intracellular Ca^2+^ that triggers the release of insulin^11^. Recently, glucose-induced electrical activity in β-cells has also been shown to depend on intracellular Cl^−^ concentration^12^, indicating the existence of an additional anionic mechanism; however, the responsible Cl^−^ channel remains unidentified. As CFTR is a cAMP/PKA-dependent Cl^−^ channel^13^ known to be gated by intracellular ATP^14^,^15^,^16^, which is metabolized from glucose taken up by the cell^17^, its expression in β-cells prompted us to hypothesize that CFTR might be sensitive to glucose and thus its activation by glucose could contribute to the glucose-induced electrical activities required for insulin secretion in the β-cell. We undertook the present study to test this hypothesis. The results show that glucose-induced whole-cell currents, membrane depolarization, electrical bursts or action potentials, Ca^2+^ oscillations and insulin secretion in β-cells are dependent on CFTR, indicating a previously unrecognized essential role of CFTR in the regulation of insulin secretion.

## Results

### Glucose-sensitive CFTR-mediated Cl^−^ currents in β-cells

**Figure 1:**
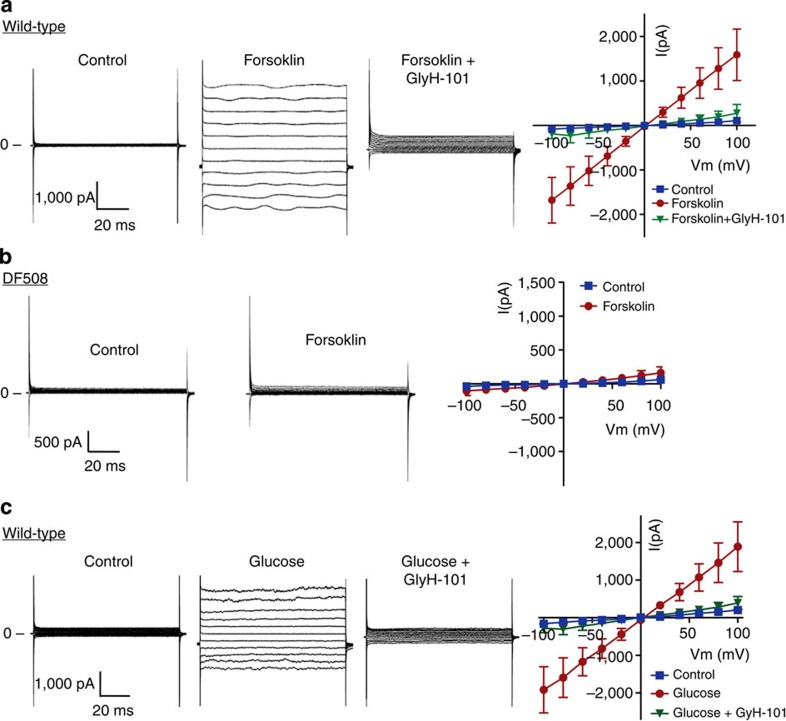
CFTR Cl^−^ currents in mouse pancreatic islet β-cells and its activation by glucose. (**a**,**b**) Whole-cell Cl^−^ currents recorded with CsCl pipette solution in CFTR wild-type (**a**) or DF508 mutant (**b**) β-cells before (control) and 5 min after the addition of forskolin (10 μM) and 3 min after CFTR inhibitor glyH-101 (10 μM) with corresponding I-V curves (*n*=3). (**c**) Whole-cell Cl^−^ current recorded with NMDG-Cl pipette and bath solution in wild-type mouse β-cells before (control) and 10 min after the addition of glucose (10 mM), and subsequently 3 min after glyH-101 (10 μM) with corresponding I-V curves (*n*=3). Pulse protocol: 20 mV steps from −100 mV to +100 mV for 100 ms. Extracellular Cl^−^ concentration ([Cl^−^]_o_)=142 mM; Intracellular Cl^−^ concentration ( [Cl^−^]_i_ )=150 mM; equilibrium potential for Cl^−^ (ECl)=1.4 mV. Data are shown as mean±s.e.m.

Using the patch-clamp technique, we examined CFTR whole-cell currents in RINm5F β-cell line and primary cultures of β-cells from wild-type and mutant mice carrying DF508, the most common CFTR mutation in CF^18^. When potassium is replaced by caesium in the pipette solution, we detected a time- and voltage-independent whole-cell current in the wild-type β-cells (Fig. 1a) or RINm5F cells (Supplementary Fig. 1a) in response to an adenylyl cyclase activator, forskolin (10 μM), with linear I-V relationship characteristic of CFTR^19^, which could be inhibited by the CFTR inhibitor, glyH-101 (10 μM). However, no significant forskolin-induced currents were observed in DF508 β-cells (Fig. 1b), suggesting that the forskolin-induced Cl^−^ currents in the wild-type β-cells were mediated by CFTR. Interestingly, currents with similar characteristics could also be activated by glucose (10 mM) in primary β-cells (Fig. 1c) with Cl^−^ as the major permeant ion in the bath and pipette solutions. We noticed that it took a longer time (10–15 min) for the cells to respond to glucose than to forskolin (3–5 min), which may reflect glucose metabolism before CFTR activation in contrast to direct activation of cAMP/PKA by forskolin. Overexpressing wild-type CFTR, but not DF508 CFTR, in Chinese hamster ovary (CHO) cells also gave rise to a glucose-induced whole-cell current, which can be inhibited by CFTRinh-172 (10 μM, Supplementary Fig. 1b,c). The observed sensitivity of CFTR to glucose, together with the reported gating of CFTR by ATP^14^, suggests its possible involvement in regulating insulin secretion in pancreatic islet β-cells.

### CFTR contributes to β-cell resting membrane potential

**Figure 2:**
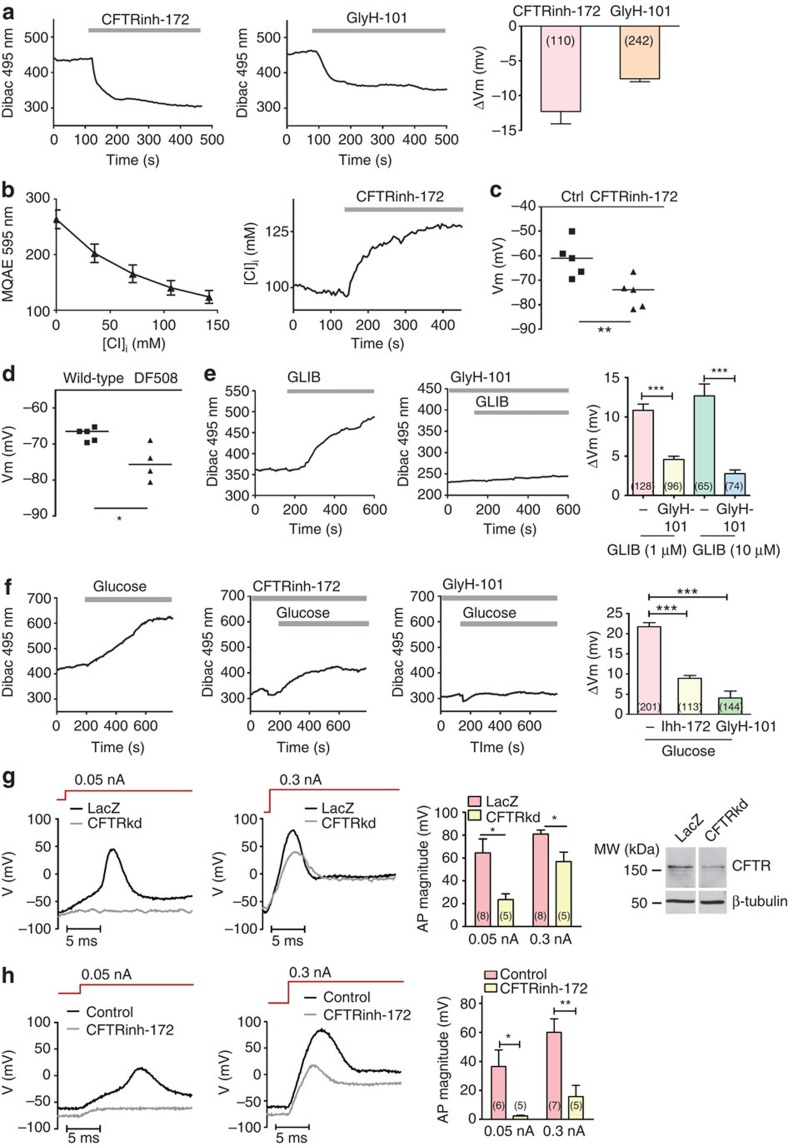
Involvement of CFTR-medicated Cl^−^ efflux in maintaining resting membrane potential of β-cells. (**a**) CFTR inhibitors, CFTRinh-172 (10 μM) and glyH-101 (10 μM), induced membrane hyperpolarization in RINm5F cells assessed by voltage-sensitive fluorometric measurement with Dibac on RINm5F cells. Number of measurements is shown in each column. (**b**) Intracellular chloride measurement with MQAE chloride-sensitive fluorescent dye in RINm5F cells (left panel: calibration curve, right panel: time course of changes in [Cl^−^]_i_). Application of CFTRinh-172 (10 μM) led to elevation of [Cl^−^]_i_ by 25.9±1.3 mM (*n*=36). (**c**) Membrane potential measurement by patch-clamp in RINm5F cells. CFTRinh-172 (10 μM) led to hyperpolarization of the membrane. **P<0.01, *t*-test. The experiment was repeated five times. (**d**) Membrane potential of β-cells measured by patch-clamp. Freshly isolated β-cells from DF508 mutant mice (*n*=4) had more negative resting membrane potential than that from wild-type mice (*n*=5). *P<0.05, *t*-test. (**e**) K_ATP_ channel inhibitor glibenclamide (GLIB, 1 μM) depolarized the membrane (left), whereas in the presence of glyH-101 (10 μM), GLIB failed to induce depolarization (right) in RINm5F cells. Number of measurements is shown in each column of the summary chart. ***P<0.001, one-way analysis of variance (ANOVA). (**f**) Glucose (10 mM) induced depolarization by about 20 mV in RINm5F cells. Pretreatment with CFTRinh-172 (10 μM) and glyH-101 (10 μM) inhibited the glucose-induced depolarization. Number of measurements is shown in each column. ***P<0.001, one-way ANOVA. (**g**,**h**) Recording of action potentials evoked by current injection in RINm5F cells; (**g**) Knockdown of CFTR (CFTRkd) decreased the action potential evoked by 0.05 and 0.3 nA currents, as compared with LacZ control, with number of experiments shown in data bars. *P<0.05, *t*-test. Western blots show the protein level of CFTR after knockdown. Uncropped immunblot is shown in Supplementary Fig. 8. (**h**) CFTRinh-172 (10 μM) completely abolished the action potential evoked by 0.05 nA current stimulus and partially abolished the action potential evoked by 0.3 nA current stimulus with number of experiments shown in corresponding data bars. *P<0.05, **P<0.01, *t*-test. [Cl^−^]_o_=142 mM (**a**–**h**). [Cl^−^]_i_=98 (**a**–**f**) and 150 (**g**,**h**) mM. ECl= −9.8 (**a**–**f**) and 1.4 (**g** and **h**). Data are shown as mean±s.e.m.

It has been well established that K_ATP_ channel has a key role in maintaining the resting membrane potential of the islet β-cell at a relatively negative level^20^ and that its inactivation by glucose results in membrane depolarization, leading to activation of voltage-sensitive Ca^2+^ channel and subsequent Ca^2+^ oscillations responsible for insulin secretion^10^,^21^. We then asked whether CFTR, as a Cl^−^ channel expressed in the β-cell, also affects the membrane potential. Using a voltage-sensitive dye, Dibac, we found that CFTR inhibitors, both CFTRinh-172 (10 μM) and glyH-101 (10 μM), could induce membrane hyperpolarization in RINm5F cells in the absence of any stimulation (Fig. 2a), indicating that CFTR may mediate a Cl^−^ efflux under basal condition. To further test this, we measured intracellular Cl^−^ concentration ([Cl^−^]_i_) using a Cl^−^ sensitive dye, MQAE. The application of CFTRinh-172 (10 μM) induced an increase in [Cl^−^]_i_ by 25.9±1.3 mM (Fig. 2b), indicating that CFTR indeed mediates Cl^−^ efflux under basal condition, which may maintain a relatively depolarized membrane potential in the β-cell at rest. This was further confirmed by patch-clamp results showing significantly more hyperpolarized resting membrane potentials in CFTRinh-172 (10 μM)-treated RINm5F cells (−75.2±2.8 mV, Fig. 2c) or DF508 β-cells (−75.3±2.5 mV, Fig. 2d) as compared with the vehicle control (−61.3±3.4 mV, Fig. 2c) or the wild-type (−67.4±0.8 mV, Fig. 2d), respectively. Of note, the averaged [Cl^−^]_i_ of RINm5F cells was determined by MQAE measurements to be at 97.7±6.6 mM, which, with the extracellular Cl^−^ concentration ([Cl^−^]_o_) of 142 mM in the bath, gave rise to an estimated Cl^−^ equilibrium potential (E_Cl_) around −9.8 mV, according to the Nernst equation. The less negative value of E_Cl_ than the observed resting potential (around −65 mV) suggests a Cl^−^ efflux from RINm5F cells at rest, consistent with the measured results (Fig. 2a–d).

We further examined the interplay between CFTR and K_ATP_ in determining the membrane potential of the β-cell. Application of glibenclamide (1–10 μM), a well-known diabetes drug that promotes insulin secretion by inhibiting K_ATP_^22^, to RINm5F cells depolarized the membrane as expected; however, in the presence of glyH-101 (10 μM), the glibenclamide-induced membrane depolarization was significantly reduced (Fig. 2e). Similarly, glucose (10 mM), which is known to inhibit K_ATP_ channel in the β-cell by elevating intracellular ATP level^21^, could result in membrane depolarization in RINm5F cells; however, the glucose-induced membrane depolarization could be blocked by CFTR inhibitors (Fig. 2f). These results suggest that the inhibition of CFTR could sufficiently hyperpolarize the membrane to counter the membrane depolarization induced by inhibition of K_ATP_ either by glibenclamide or glucose. To further support this notion, we applied electrical stimulus to RINm5F cells to evoke action potentials using the patch-clamp technique and found that in the presence of CFTRinh-172 (10 μM) (Fig. 2h) or when CFTR was knocked down (by about 65%) (Fig. 2g), it required a greater injecting current, 0.3 nA, instead of 0.05 nA in the controls, to elicit an action potential. Taken together, these results suggest that in addition to K_ATP_, CFTR also has an important role in determining the resting membrane potential of the β-cell, inhibition or defect of which results in membrane hyperpolarization.

### CFTR contributes to the glucose-induced action potentials

**Figure 3:**
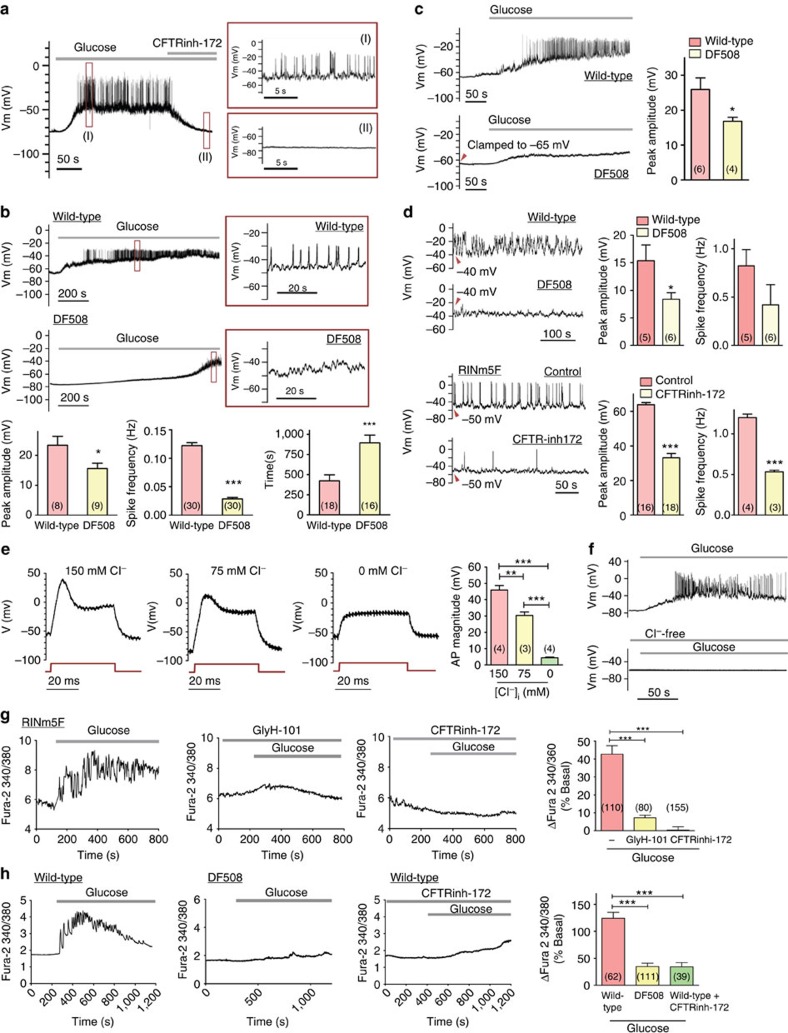
Involvement of CFTR-mediated Cl^−^ efflux in glucose-induced electric spikes and calcium oscillations in β-cells. (**a**) Glucose (10 mM) elicited a slow membrane depolarization superimposed with bursts of action potentials (spikes) in isolated mouse islet β-cells (enlarged in I), which could be abolished by CFTRinh-172 (10 μM) (enlarged in II). *n*=4. (**b**) Islet β-cells from DF508 mice required a significantly longer time to reach the threshold for spike generation and had a smaller magnitude and frequency of glucose (10 mM)-induced spikes than that from the wild types. Number of measurements is shown in each column *P<0.05, ***P<0.001, *t*-test. (**c**) When the membrane potential was clamped to −65 mV similar to that observed in wild-type, DF508 β-cells (lower panel) still exhibited a reduced magnitude of the glucose-induced spike than that from wild type (upper panel). Number of measurements is shown in each column. *P<0.05, *t*-test. (**d**) Upper panel: when the membrane potential was clamped to depolarization voltage (−40 mV), electric spikes generated in wild-type β-cells exhibited higher magnitude and frequency than that in DF508. Lower panel: when the membrane potential was elevated to a depolarized level, −50 mV, CFTRinh-172 (10 μM) decreased the spike frequency and amplitude in RINm5F cells. Number of measurements is shown in each column. *P<0.05, ***P<0.001, *t*-test. [Cl^−^]_o_=142 mM; [Cl^−^]_i_=150 mM and ECl=1.41 mV (**a**–**d**). (**e**) Intracellular chloride concentration-dependent action potential in RINm5F cells. [Cl^−^]_o_=142 mM; ECl=1.4, −16.5 and −∞ with [Cl^−^]_i_ at 150, 75 and 0 mM, respectively. *n*=4. **P<0.01; ***P<0.001, one-way analysis of variance (ANOVA). (**f**) When Cl^−^ was removed from the pipette solution, the glucose-induced electric spikes were almost completely abolished in RINm5F cells. (**g**) Effect of CFTRinh-172 (10 μM) and glyH-101 (10 μM) on glucose (10 mM)-induced intracellular Ca^2+^ and oscillations measured by Fura-2 in RINm5F cells. Number of measurements is shown in each column. ***P<0.001, one-way ANOVA. (**h**) Glucose (10 mM)-induced Ca^2+^ increase and oscillations in β cells isolated from the wild-type and DF508 mice with or without CFTRinh-172 (10 μM). Number of measurements is shown in each column. ***P<0.001, one-way ANOVA. Data are shown as mean±s.e.m.

The action potentials or electric spikes leading to insulin secretion in the β-cell are generally believed to be contributed by Ca^2+^ and Na^+^ influxes^23^. However, it has been reported that the spikes also depend on intracellular Cl^−^ (ref. 12). As shown in Fig. 2g,h, CFTR inhibition or knockdown significantly reduced the magnitude of the action potentials. We thus asked whether CFTR could also contribute to the action potentials in β-cells, in addition to the resting membrane potential. As shown in Fig. 3a, glucose (10 mM) elicited a slow membrane depolarization superimposed with bursts of action potentials (electric spikes) in β-cells isolated from mice, which could be abolished by subsequent addition of CFTRinh-172 (10 μM). Similarly, the magnitude of the glucose-induced electric spikes in β-cells isolated from DF508 mice was significantly reduced (15.6±1.7 mV) as compared with the wild-type (23.4±3.0 mV), and so was the frequency of the spikes (from 0.122 Hz in wild-type to 0.028 Hz in DF508) (Fig. 3b). Moreover, the time duration for the membrane potential to reach the threshold for the generation of the electric spikes in DF508 (894.5±96.7 s) was significantly longer than that in wild-type β-cells (423.4±73.4 s) (Fig. 3b), consistent with the more hyperpolarized resting membrane observed in CFTR defective or inhibited β-cells (Fig. 2c,d). The attenuation of electric spikes in CFTR inhibited/defective β-cells could also be due to the hyperpolarized resting membrane potential that prevents firing of action potentials or the spikes. To test this, we injected currents to elevate the membrane potential of DF508 cells to the averaged resting membrane potential level observed in wild-type cells, −65 mV. However, under this condition, the spikes evoked by glucose (10 mM) in DF508 cells were still significantly smaller than that of wild-type cells (Fig. 3c), indicating possible contribution of CFTR to the electric spikes independent of its effect on the resting membrane potential. Furthermore, injecting currents to elevate the membrane potential of both wild-type and DF508 β-cells to the same depolarized level, −40 mV, evoked electric spikes in the absence of glucose in wild-type cells with a magnitude significantly greater than that observed in DF508 β-cells (Fig. 3d), further demonstrating the direct involvement of CFTR in the generation of the electric spikes. Similar results were obtained from RINm5F cells showing that the depolarization (−50 mV)-induced electric spikes in the absence of glucose could be attenuated, both in magnitude and frequency, by CFTRinh-172 (10 μM, Fig. 3d). These results suggest that during the rapid changes of membrane potentials that constitute the electrical spikes of β-cells, there could be transient inward currents that are contributed by CFTR-mediated Cl^−^ efflux. To further test this, we examined the Cl^−^ dependence of the action potential in β-cells. Indeed, in the patch-clamp experiments on RINm5F cells, when Cl^−^ in the pipette solution was varied, the magnitude of the action potential generated by injecting 0.3 nA current was also varied, with the highest observed at 150 mM, intermediate at 75 mM and almost no action potential at 0 mM (Fig. 3e), indicating the dependence of the action potentials on intracellular Cl^−^, or Cl^−^ efflux. When Cl^−^ was removed from the pipette solution, the glucose-induced electric spikes were almost completely abolished in RINm5F cells (Fig. 3f), similar to that observed in CFTR DF508 β-cells (Fig. 3c). Taken together, the dependence of the β-cell action potentials on both CFTR and intracellular Cl^−^ suggests that the glucose-induced electric spikes in β-cells may be altered when Cl^−^ currents flow through CFTR.

### Involvement of CFTR in glucose-induced Ca^2+^ mobilization

It is well-known that glucose-induced action potentials are coupled to the Ca^2+^ influx and oscillations required for insulin release from the β-cell. By measuring intracellular Ca^2+^, we observed that both CFTRinh-172 (10 μM) and glyH-101 (10 μM) could significantly attenuate the Ca^2+^ increase and abolish the Ca^2+^ oscillations induced by glucose (10 mM) in RINm5F cells (Fig. 3g). The Ca^2+^ response to glucose in β-cells from CFTR DF508 mice was also significantly attenuated as compared with that observed in the wild-type (Fig. 3h). Similar reduction in Ca^2+^ response was also observed when wild-type islet cells were challenged with CFTRinh-172 (Fig. 3h). The number of cells responded to glucose (10 mM) with intracellular Ca^2+^ increase was greatly reduced in DF508 (10.3±2.8%) or in the presence of CFTRinh-172 (8.1±4.2%) as compared with that of wild-type control (69.3±15.8%, Supplementary Fig. 2). These results are consistent with the demonstrated role of CFTR in mediating the glucose-induced action potentials, and thus, affecting the electrically coupled Ca^2+^ response.

### CFTR regulates insulin secretion

**Figure 4:**
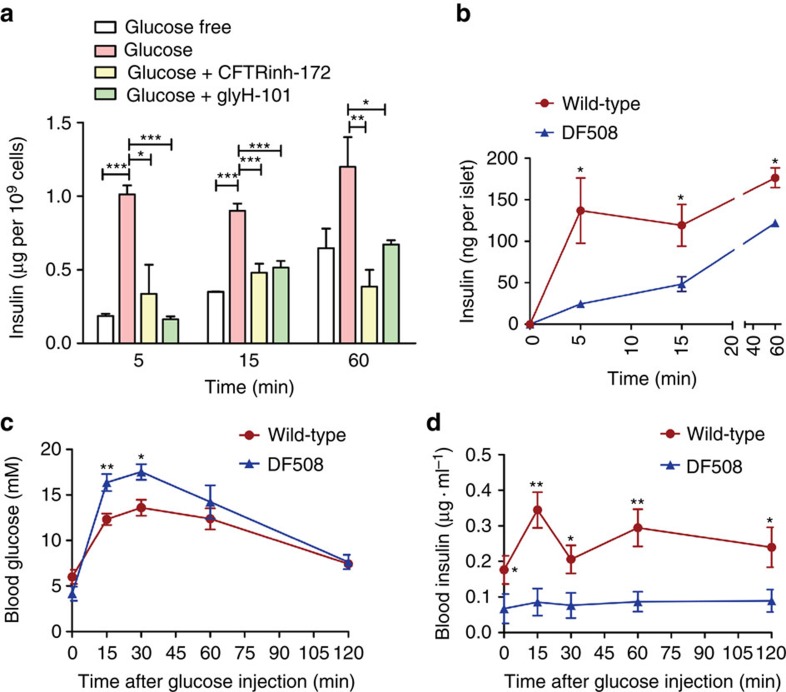
Involvement of CFTR in glucose-induced insulin secretion. (**a**) Effect of CFTRinh-172 and glyH-101 on glucose-induced insulin secretion of islets isolated from mice. CFTRinh-172 (10 μM) and glyH-101 (10 μM) significantly reduced insulin secretion 5, 15 and 60 min after glucose (10 mM) challenge. The experiment was repeated three times. *P<0.05, ***P<0.001, one-way analysis of variance. (**b**) Reduced glucose (10 mM) -induced insulin secretion from CFTR DF508 islets as compared with the wild-type. *n*=3–4, *P<0.05, *t*-test. (**c**,**d**) Time-course change in blood glucose (**c**) and insulin (**d**) after glucose injection (intraperitoneal 2 g kg^−1^ body weight) in DF508 and the wild-type mice. *P<0.05; **P<0.01. *n*=6, *t*-test. Data are shown as mean±s.e.m.

We next asked whether CFTR has a sufficiently important role in regulating insulin secretion. ELISA measurement on RINm5F cell culture medium showed that both CFTR inhibitors significantly reduced insulin secretion 5 and 15 min after glucose (10 mM) challenge, but without significant effect on the insulin levels 60 min after glucose challenge (Supplementary Fig. 3). Similarly, the glucose-induced insulin secretion by freshly isolated mouse islets 5, 15 and 60 min after glucose (10 mM) challenge was also significantly inhibited by the two CFTR inhibitors, with greater reductions observed at the early time point, 5 min, compared with 60 min (Fig. 4a). Similar results were also obtained with glibenclamide-induced insulin secretion (Supplementary Fig. 4). Isolated islets from DF508 mice also exhibited significant reductions in insulin secretion compared with the wild-type with greater difference observed at 5 min (around 80%) than that at 60 min (around 30%) after glucose challenge (Fig. 4b), which is consistent with the lack of the first phase response to glucose challenge in CFRD patients^4^,^24^. We also performed *in vivo* glucose tolerance test. An injection of glucose (2 g kg^−1^ body weight) was made in mice after overnight fasting. As shown in Fig. 4c, in both DF508 and wild-type mice, the blood glucose levels arose within 30 min after the glucose injection, which went back down to the initial level gradually in 2 h. Although there was no significant difference in the initial glucose levels between the two, DF508 mice exhibited significantly higher blood glucose level than that of the wild types 15 and 30 min after the glucose injection (Fig. 4c). Moreover, the blood insulin level in DF508 mice was found persistently lower than that in the wild types during the whole course of the test (Fig. 4d). These *in vivo* results further indicate an important role of CFTR in insulin secretion.

### Rescue of DF508 defects by corrector VX-809

**Figure 5:**
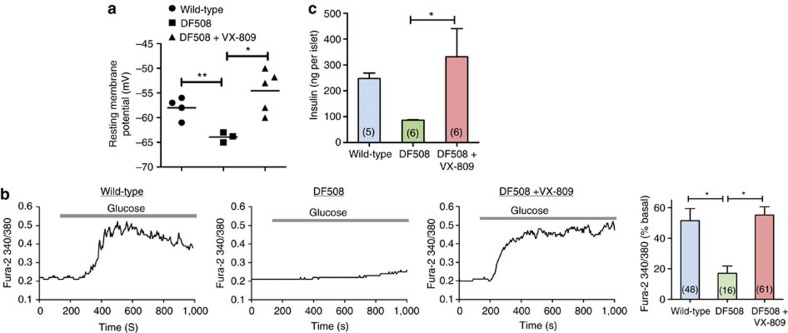
Rescue of DF508 defects by corrector VX-809. Effect of VX-809, a corrector of DF508 CFTR, in rescuing DF508 defects, resting membrane potential (**a**, by patch-clamp), Ca^2+^ response (**b**, by Fura-2) and insulin secretion (**c**). Islets/β-cells isolated from DF508 mice were pretreated with VX-809 (10 μM) for 48 h before the experiments. DMSO (0.1% v/v) was also applied to both wild-type and DF508 islets/β-cells as vehicle control for comparison. Number of measurements is shown in each column. *P<0.05,**P<0.01, one-way analysis of variance. Data are shown as mean±s.e.m.

We then attempted to rescue the DF508 β-cell defects using the newly discovered corrector of DF508, VX-809, which corrects mis-folding of the mutant CFTR protein and thus prevents its rapid degradation^25^,^26^. After incubation with VX-809 (10 μM) for 48 h, DF508 β-cells showed significantly elevated membrane potential levels as compared with the dimethyl sulphoxide (DMSO)-treated control DF508 β-cells (Fig. 5a). Also, the VX-809-treated DF508 β-cells showed significantly higher glucose-induced Ca^2+^ responses than the DMSO-treated controls, which was also comparable to that of wild-type β-cells (Fig. 5b). More importantly, treatment of DF508 islets with VX-809 increased the glucose-induced insulin secretion in a concentration-dependent manner (1–10 μM), which was significantly higher than that of the DMSO-treated DF508 islets and comparable to the levels observed in the wild type (Fig. 5c & Supplementary Fig. 5a). Of note, VX-809 (0.01–100 μM) showed no effect on cell variability (Supplementary Fig. 5b) and the VX-809-induced increase in insulin release could be abolished by nifedipine (10 μM, Supplementary Fig. 5c), a blocker of voltage-dependent Ca^2+^ channels known to inhibit insulin release^27^, ruling out possible toxic effect of VX-809.

## Discussion

Taken together, the present results have revealed a previously unrecognized contribution of CFTR to the electrophysiological properties of pancreatic β-cells, which are fundamentally important for insulin secretion. CFTR appears to be open basally in the β-cell and mediates the Cl^−^ efflux maintaining the resting membrane potential at a level less hyperpolarized than that if the membrane potential is entirely determined by K_ATP_. This is evidenced by the observation that when CFTR is inhibited or defective, the membrane would be hyperpolarized such that inhibiting K_ATP_ by glibenclamide or glucose fails to depolarize the membrane (Fig. 2e,f). The present results also suggest that basally open CFTR can be further activated by glucose, with Cl^−^ efflux affecting the glucose-induced membrane depolarization and bursting action potentials, both in magnitude and frequency, which are coupled to Ca^2+^ oscillations and subsequently insulin secretion. Of note, it has been reported that the depolarized level brought about by closure of K_ATP_ channel cannot reach the threshold to activate the voltage-dependent Ca^2+^ channel^10^. This implies that other channel(s), such as CFTR as demonstrated presently, may be involved in bringing β-cell membrane to a depolarized state necessary for initiating the sequence of events leading to insulin secretion. The observed activation of CFTR by glucose (Fig. 1c) and the fact that CFTR’s opening depends on ATP binding to its NBD domains^14^ make CFTR a perfect candidate, in addition to K_ATP_, in sensing glucose change and thus regulating insulin secretion. This notion is further supported by the observation that overexpression of CFTR in RINm5F cells with intact or knocked-down K_ATP_ increased the glucose-induced membrane voltage changes, Ca^2+^ oscillations and insulin secretion (Supplementary Fig. 6). However, it remains unresolved whether CFTR is directly gated by glucose-induced ATP changes, or by glucose-dependent kinases^28^,^29^ that may phosphorylate CFTR^30^. The exact mechanism underlying CFTR activation by glucose awaits further investigation. Interestingly, a previous study using voltage-clamp technique has shown that the orientation of current during glucose-induced depolarization was altered by varying E_Cl_ in rat pancreatic β-cells^12^, consistent with the involvement of a Cl^−^ conductance during the process as observed in the present study. Although volume-regulated anion channel was implicated, the molecular entity of the responsible channel was not identified. Of note, although a granular Cl^−^ transporter, ClC-3, has also been implicated in insulin-secretion^31^,^32^, its exact role is currently under intense debate^33^. The granular localization of ClC-3 also makes it unlikely that it would contribute significantly to the glucose-induced membrane depolarization observed in the present study.

What are the pathophysiological consequences to the defect of CFTR in pancreatic β-cell? A more hyperpolarized membrane potential at rest and the attenuated glucose-induced action potential amplitudes due to inhibition or defective CFTR, as demonstrated in the present study (Figs 2 and 3), may render the β-cell a slower response to glucose challenge, that is, it takes longer to reach the threshold of the glucose-elicited action potential from a more hyperpolarized membrane potential compared with a more depolarized membrane potential if CFTR is intact. It should be noted that the first phase of insulin secretion in response to glucose is known to be crucially associated with membrane voltage changes, whereas the second phase is reported to be largely driven by Ca^2+^ channels that are less dependent on membrane voltage^34^. This explains why CFRD patients lack the first phase of insulin secretion^24^, as the secretory granule mobilization associated with the first phase of insulin secretion is known to be dependent on voltage-gated Ca^2+^ channels^35^. Interestingly, in contrast to the current belief that insulin insufficiency in CFRD is mainly due to destruction of the pancreatic islets^6^, our H&E examination revealed no significant difference in pancreatic islet morphology between CFTR wild-type and DF508 mice (Supplementary Fig. 7), indicating that the observed defect in insulin secretion in CF may not be caused by structural alteration of the islets. Of note, CFRD may start in CF patients at ages as children or juveniles with absence of islet destruction^5^,^36^. Thus, a loss of islet cells could be at most a long-term effect in CFRD patients^36^. The present findings provide an alternative explanation for the pathogenesis of CFRD, abnormal β-cell electrophysiological properties underlying insulin secretion due to the defect in CFTR Cl^−^ channel function. The demonstrated correction of defects in DF508 β-cells by VX-809, including membrane potentials, Ca^2+^ responses and insulin secretion, further confirms an essential role of CFTR in β-cell function and insulin secretion. This notion, as well as the human relevance and therapeutic implication of the present finding, is further supported by a most recent study conducted in CFRD patients showing improved insulin secretion with a corrector for another mutation of CFTR^37^. Of note, over 1,900 mutations in *CFTR* have been identified in humans, which do not necessarily lead to phenotypic CF. Thus, the presently demonstrated critical role of CFTR in regulating insulin secretion suggests a plausible cause of idiopathic diabetic conditions due to a ‘mild’ non-CF mutation or abnormal CFTR expression/function in general population other than CF, although such a mutation has not been found so far in genome-wide association study analyses in the normal population with diabetes^38^. The present findings warrant future investigation into this possibility.

## Methods

### Animals

C57 and DF508 mice were purchased from the Laboratory Animal Service Centre of the Chinese University of Hong Kong. All animal experiments were conducted in accordance with the university guidelines on animal experimentation, and approval by the Animal Ethics Committee of the Chinese University of Hong Kong was obtained for all related procedures.

### Cell culture

RINm5F cells purchased from ATCC were cultured in RPMI 1640 supplemented 10% FBS, 100 IU ml^−1^ penicillin and 100 μg ml^−1^ streptomycin. The glucose responsiveness of the RINm5F cells used in the present study was confirmed by immediate electrical, Ca^2+^ and insulin-releasing responses to the addition of glucose in culture media after 1-h glucose-free incubation. CHO cells were cultured in DMEM (high glucose) with 10% FBS.

### Islet isolation

C57 male mice, CFTR wild-type or DF508 mutant mice at the age of 12–14 weeks were killed by CO_2_ inhalation. HBSS containing 1 mg ml^−1^ Type XI collagenase was injected into the pancreas via the bile duct. The pancreas was removed and incubated in 37 °C for 10 min, then transferred to 150 mm petri dish with culture medium. Islets were collected and cultured individually in 96-well plates for insulin measurement. Isolated islets were further incubated in enzyme free cell dissociation solution (Millipore, Cat. S-004-B) for 5 min and dispersed into a single cell for patch-clamp and calcium measurement^39^.

### Whole-cell patch-clamp recording

RINm5F and single islet cells were cultured on coverslips for 3 days before patch-clamp recording. Borosilicate glass-made patch pipettes (Vitrex, Modulohm A/S, Herlev, Denmark), were pulled with micropipette puller (P-97, Sutter Instrument Co., USA) to a resistance of 5–7 MΩ after being filled with pipette solution. Ionic current was recorded with a data acquisition system (DigiData 1322A, Axon Instruments) and an amplifier (Axopatch-200B, Axon Instruments, Foster City, CA, USA). The command voltages were controlled by a computer equipped with pClamp Version 9 software.

For CFTR function experiment, cells were bathed in solution (in mM): NaCl 130, KCl 5, MgCl_2_ 1, CaCl_2_ 2.5, Hepes 20 with D-manitol compensated for osm 310 (pH 7.4); pipettes were filled with solution (in mM): CsCl 101, EGTA 10, HEPES 10, TEACl 20, MgATP 2, MgCl_2_ 2, glucose 5.8, PKA subunit 100 U ml^−1^ with D-manitol compensated for osm 290 or NMDG-Cl 140, HEPES 20, EGTA 10, MgSO_4_ 1 with D-manitol compensated for osm 290. When the whole-cell Giga seal was formed, the capacitance of cell was measured. Islet cells with capacitance larger than 6 pF were considered to be β-cells^40^. The whole-cell current was obtained by voltage clamp with the commanding voltage elevated from −100 mV to +100 mV with 20 mV increment^41^.

For action potential recording, the pipettes were filled with solution (in mM): KCl 138, NaCl 10, MgCl_2_ 1 and HEPES 10 with D-manitol compensated for osm 290. In Cl^−^ free experiments, Cl^−^ was replaced by gluconate. Action potential is evoked by 0.05 or 0.3 nA current injection.

### Membrane potential (Vm) measurement

Before Vm measurement, cells were washed with bath solution (Margo-Ringer glucose-free solution) containing (mM): NaCl 130, KCl 5, MgCl_2_ 1, CaCl_2_ 2.5, Hepes 20 with D-manitol compensated for osm 285 (pH 7.4), then transferred to a mini chamber containing 1 ml bath solution. Then the cells were loaded with the voltage-sensitive dye DiBAC4(3) (2.5 μM). The chamber was mounted on to a fluorescence microscope (Eclipse Ti, Nikon, Tokyo, Japan) and fluorescence (495/520 nm excitation/emission) was monitored at room temperature. An increase in fluorescence corresponds to depolarization of the membrane potential, whereas a decrease corresponds to hyperpolarization of the membrane potential. To calibrate the change in fluorescence intensity with membrane voltage changes, incremental concentrations of potassium gluconate (5, 10, 20, 40 and 60 mM) were added with valinomycin (2 μM) to the bath solution. The resulting changes in fluorescence intensity were recorded and subtracted from the background intensity. The expected changes in membrane potential induced by increasing extracellular K^+^ concentrations were calculated using the Nernst equation assuming an intracellular K^+^ concentration of 120 mM. The calibration curve was obtained by fitting the potassium gluconate-induced fluorescence intensity change (% basal level) to the calculated membrane potential change (mV), which was later used to obtain the membrane potential changes^42^,^43^.

### Intracellular calcium measurement

Before calcium measurements, cells were washed with the same bath solution as used for Vm measurement. Then cells were loaded with Fura-2-AM (3 μM) in the bath solution at 37 °C for 30 min. The coverslip was transferred to a mini chamber containing 1 ml bath solution and mounted on to a fluorescence microscope (Eclipse Ti, Nikon, Tokyo, Japan). Fluorescence was alternatively excited by dual wave length at 340 and 380 nm, and emission signals were recorded at 510 nm. Intracellular calcium change was reflected by the change in the ratio of 340/380 fluorescent signal intensity.

### Intracellular chloride measurement

Before chloride measurement, cells were washed with the bath solution and loaded with 10 mM MQAE [N-(6-methoxyquinolyl)-acetoethyl ester] at 37 °C for 30 min. Fluorescence was excited at 340 nm and emission signals were recorded at 460 nm. To calibrate the change of fluorescence intensity with the change of intracellular Cl^−^, incremental concentrations of Cl^−^ (0–105 mM) with 10 μM nigericin+10 μM tributyltin or 105 mM KSCN with 5 μM valinomycin were added to the bath solution. The calibration curve was obtained by fitting fluorescence intensity corresponding to Cl^−^ concentration.

### CFTR overexpression

Cells were plated at density of 0.25 × 10^4^ ml^−1^ 24 h before transfection. A total of 2 μg plasmid with wild-type or DF508 CFTR sequences (for CHO cells transfection, 0.3 μg GFP plasmid was added) were mixed with 10 μl SuperFect Transfection Reagent (Qiagen, Cat. No.: 301305) in 100 μl Opti-MEM Reduced Serum Medium and incubated for 15 min. The plasmid-transfection reagent mixture was added to cells in 600 μl medium. For CHO cells, after 3 h incubation, the medium with transfection reagents were replaced with growth medium. CHO cells with green florescence were used for patch-clamp experiments 24 h after transfection. For RINm5F cells, after 48-h transfection, cells were used for insulin, calcium or membrane potential measurements.

### CFTR and K_ATP_ knockdown

miRNA expression sequences targeting rat CFTR mRNA (F: 5′-TGCTGAGTAATAGCCAACATCTCTCCGTTTTGGCCACTGACTGACGGAGAGATTGGCTATTACT-3′ and R: 5′-CCTGAGTAATAGCCAATCTCTCCGTCAGTCAGTGGCCAAAACGGAGAGATGTTGGCTATTACTC-3′) were inserted into pcDNA6.2-GW/EmGFP-miR expression vector by BLOCK-iT Pol II miR RNAi Expression Vector Kits. LacZ sequences were used as negative control. A total of 6 μg plasmids were transfected into RIN-5F cells in each well of the 6-well plate by lipofectamine 2000. Whole-cell protein was extracted for western blot analysis at 72 h after transfection.

To knock down K_ATP_, siRNAs, Kir6.2 (sc-270034, Santa Cruz) or control (12935200, Invitrogen), were transfected with lipofectamine 2000 into RINm5F cells. Forty-eight hours after transfection, the medium was collected for insulin measurement and the cells were used for calcium or membrane potential measurements.

### Insulin ELISA

RINm5F cells were grown on 24-well plates. Isolated islets were cultured in 96-well plates. The cells or islets were fasted from glucose for 2 h before adding 10 mM glucose. Islets with similar size (about 100 μm diameter) were used for insulin ELISA measurement. Culture media were collected 5, 15 and 60 min after the glucose challenge. Insulin in the culture media was measured by ELISA following the manual of the manufacturer (Mercodia).

### Glucose tolerance test

Mice were deprived of food for overnight before an injection of glucose (intraperitoneal, 2 g kg^−1^ body weight) was made on each mouse. Blood were collected via the tail 0, 15, 30, 60 and 120 min after the glucose injection. Glucose and insulin levels in the collected blood were determined by glucose test strip (Bayer HealthCare LLC) and insulin ELISA kit (Mercodia) respectively.

### Statistics

Data are represented as mean±s.e.m. Two-tail unpaired Student’s *t*-tests were used for comparison between two groups. For three or more groups, data were analysed by one-way analysis of variance and Tukey’s *post hoc* test. A probability of *P*<0.05 was considered to be statistically significant.

## Author contributions

H.C.C. conceptualized; J.H.G., Y.C.R. and Y.S. designed; J.H.G., H.C., Y.C.R., X.L.Z., X.H.Z., K.L.F., L.L.T., M.K.Y., W.Q.H., X.S. and Y.W.C. was involved in the experimentation and data analysis; X.J. gave intellectual input; J.H.G., Y.C.R. and H.C.C. wrote the manuscript.

## Additional information

**How to cite this article:** Guo, J. H. *et al.* Glucose-induced electrical activities and insulin secretion in pancreatic islet β-cells are modulated by CFTR. *Nat. Commun.* 5:4420 doi: 10.1038/ncomms5420 (2014).

## Supplementary Material

Supplementary Information Supplementary Figures 1-8
