# Supplementary material for: Glucose-induced electrical activities and insulin secretion in pancreatic islet β-cells are modulated by CFTR
Source: Nat Commun. 2014 Jul 15;5:4420. doi: 10.1038/ncomms5420 (PMC4104438; doi:10.1038/ncomms5420)
Supplement: Supplementary Information — Supplementary Figures 1-8 [file ncomms5420-s1.pdf]

Supplementary Fig. 1

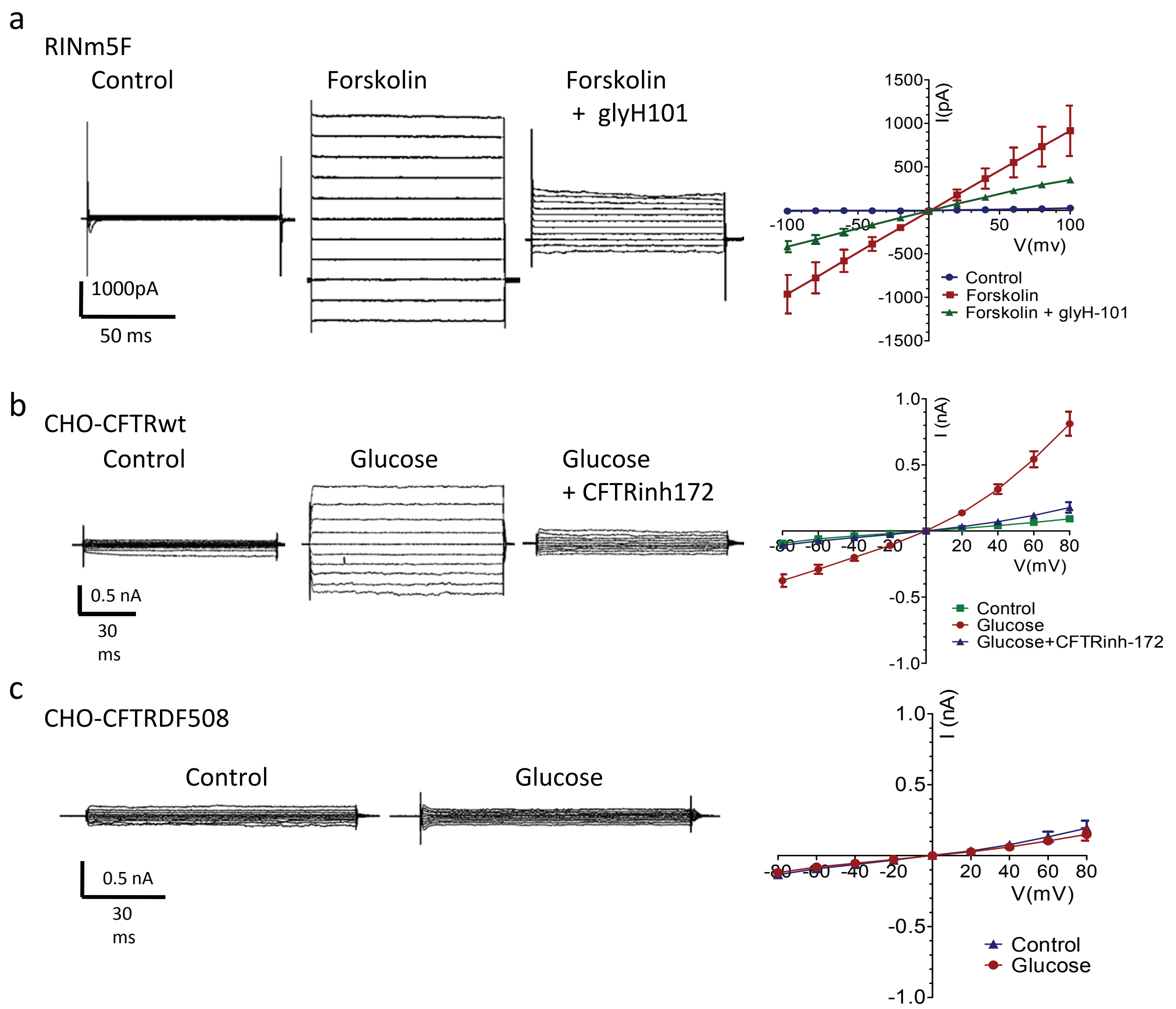

Supplementary Fig. 1 : Functional expression of CFTR in β cells and activation of CFTR by glucose in CHO cells. (a) Whole-cell Cl^- currents recorded with CsCl pipette solution from -100mV to 100mV in RINm5F cells before (control) and after challenge of 10µM forskolin and 10µM CFTR inhibitor glyH-101 with corresponding I-V curves (n=3). **(b&c)** Whole-cell Cl^- currents recorded in response to glucose in wildtype and DF508 CFTR overexpressed CHO cells. In wild type CFTR (c) expressing CHO cells, 20mM glucose directly activated whole-cell Cl^- currents with a time and voltage independent characteristic. (voltage from -100mV to 100mV with a delta of 20mV, and each step lasts 100ms), which could be blocked by CFTRinh-172 (10 µM). In DF508 mutant CFTR (d) expressing CHO cells, 20mM glucose failed to activate whole-cell Cl^- currents. Data are shown as mean ± SEM.

Supplementary Fig. 2

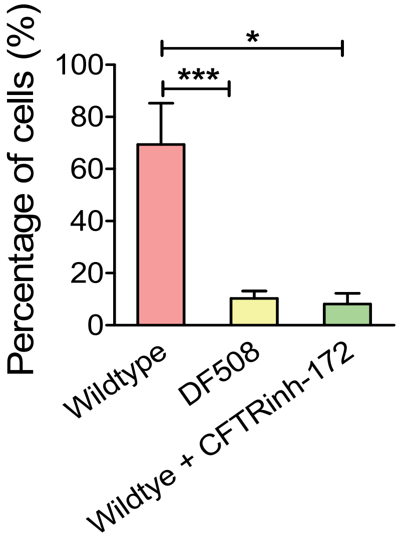

Supplementary Fig. 2 : Glucose-induced calcium response in β-cells from CFTR wildtype and DF508 mutant mice. The percentage of cells responded to glucose (10 mM) with elevated intracellular calcium was higher in wildtype islet cells than in DF508 islet cells and wildtype islet cells treated with CFTRinh-172 (10 μM). *: *P*<0.05, ***: *P*<0.001, one-way ANOVA. Data are shown as mean ± SEM.

Supplementary Fig. 3

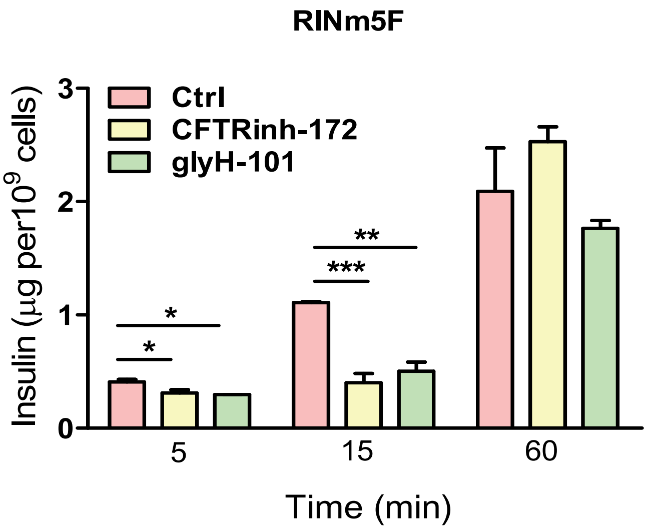

Supplementary Fig. 3 : CFTR inhibition attenuates insulin secretion in RINm5F cells. CFTR inhibitors, CFTRinh-172 (10 μM) and glyH-101 (10 μM), reduced insulin secretion 5 and 15 mins after glucose (10 mM) challenge. *: *P*<0.05, **: *P*<0.01, ***: *P*<0.001, one-way ANOVA. Data are shown as mean ± SEM.

Supplementary Fig. 4

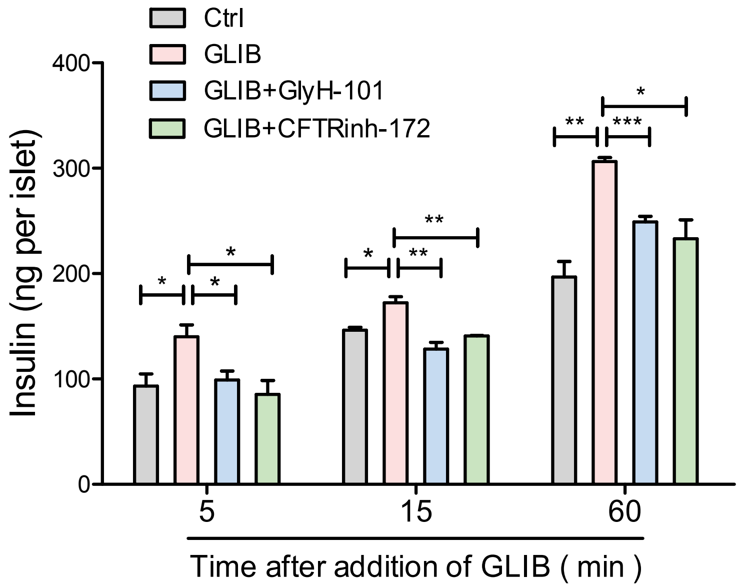

Supplementary Fig. 4 : CFTR inhibition attenuates glibenclimide-induced insulin secretion in mouse islets. Pretreatment of CFTR inhibitors, CFTRinh-172 (10 μM) and glyH-101 (10 μM), reduced insulin secretion 5 , 15 and 60 mins after Glibenclimide (10 μM) challenge. *: P<0.05, **: P<0.01, ***: P<0.001, one-way ANOVA. Data are shown as mean ± SEM.

Supplementary Fig. 5

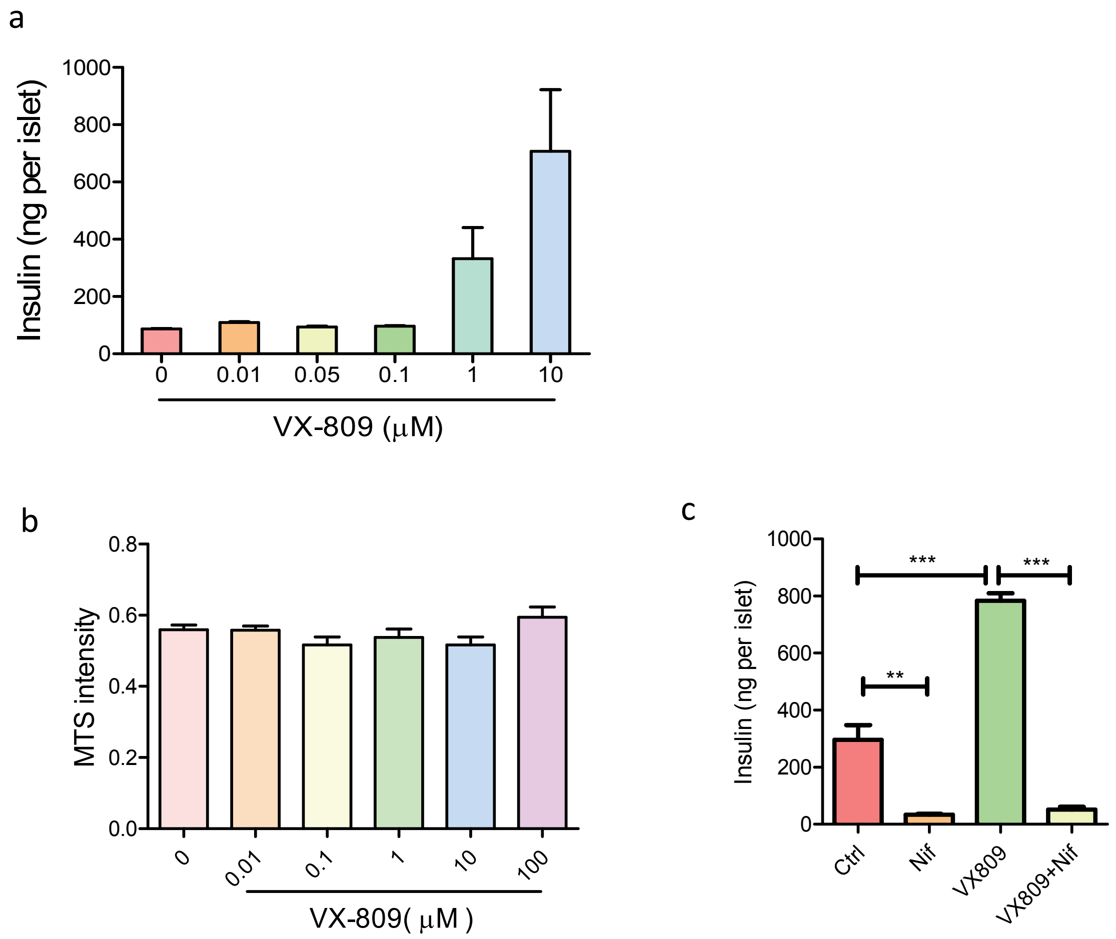

Supplementary Fig. 5 : Effect of VX-809 on insulin secretion and cell viability. a) Glucose-induced insulin secretion in islets isolated from DF508 mutant mice after treatment with different concentrations of VX-809. **b)** MTS assay show the effect of VX-809 (0.01-100 μM) on the viability of RINm5F cell. **c)** VX809 (10 μM)-induced increase in insulin secretion is abolished by nifidepine (Nif, 10 μM), a Ca^{2+} channel blocker that inhibits insulin secretion. *: *P*<0.05, **: *P*<0.01,***: *P*<0.001, one-way ANOVA. Data are shown as mean ± SEM.

Supplementary Fig. 6

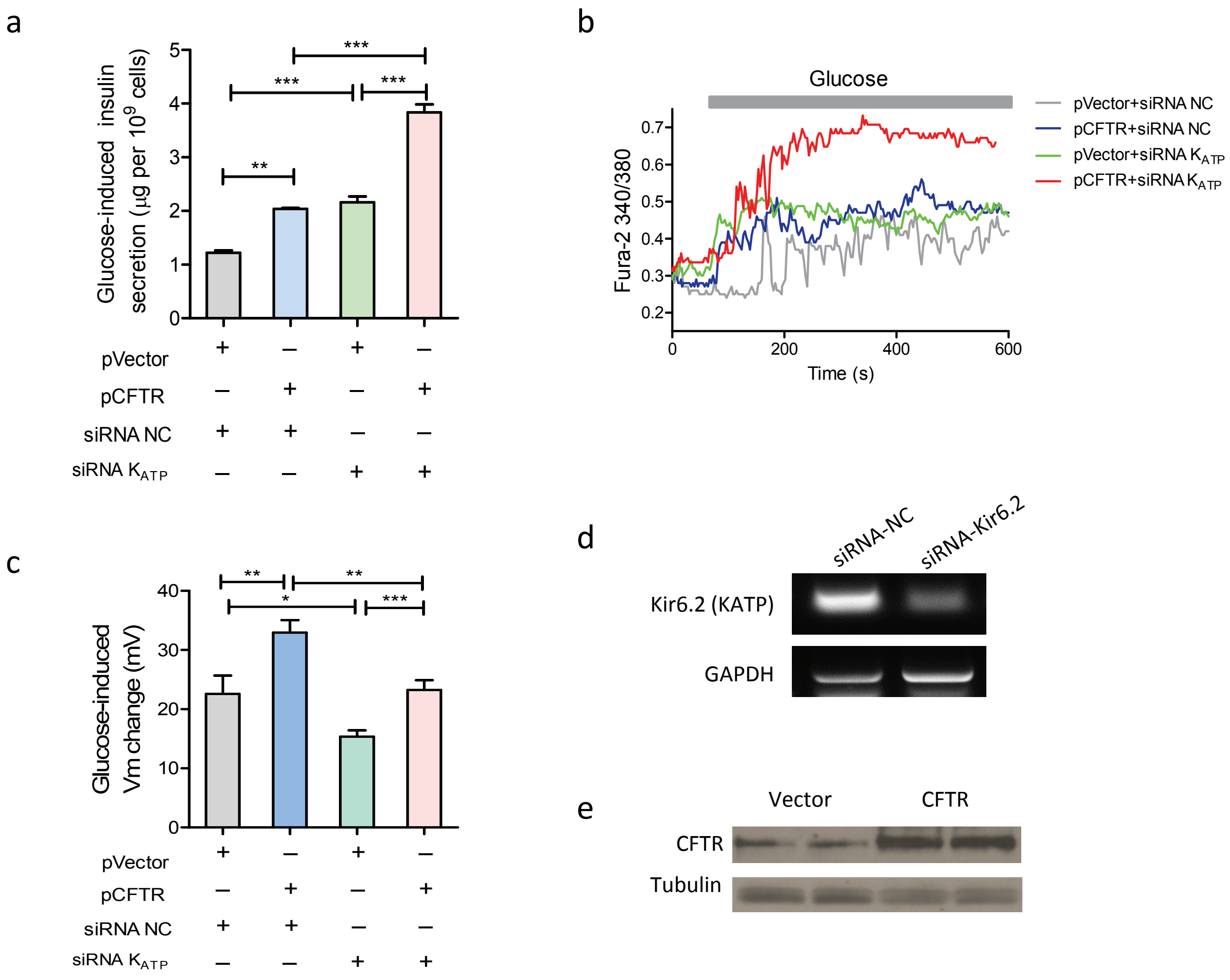

Supplementary Fig. 6 : Effect of CFTR overexpression on glucose-induced insulin secretion (a), Ca^{2+} response (b) and membrane depolarization (c) in RINm5F cells with normal and knocked-down KATP. d) Confirmation of KATP knockdown by RT-PCR (d) and CFTR overexpression by western blot (e) with siRNA targeting KATP (siRNA-Kir6.2) or non-silencing siRNAs as negative controls (siRNA-NC). *: *P*<0.05; **: *P*<0.01; ***: *P*<0.001, one-way ANOVA. Data are shown as mean ± SEM.

Supplementary Fig. 7

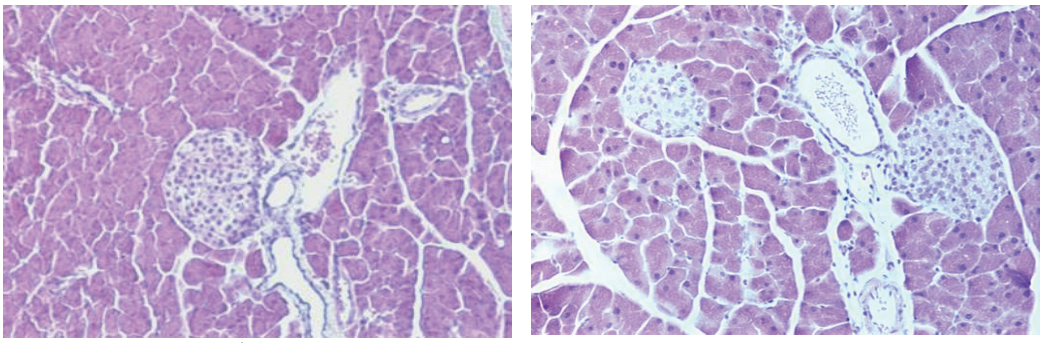

Supplementary Fig. 7 : Histology of pancreas in CFTR wildtype and DF508 mice. H-E staining shows no significant difference in pancreatic histology between wildtype and CFTR mutant mice. Data are shown as mean ± SEM.

Supplementary Fig. 8

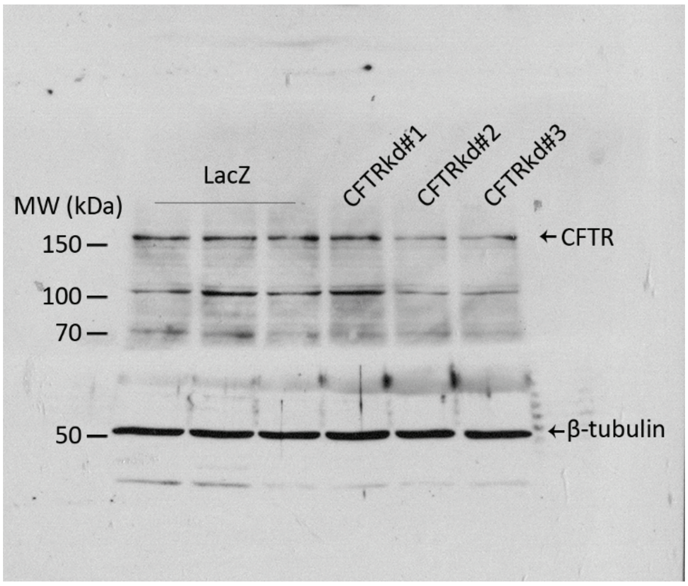

Supplementary Fig. 8: Uncropped western blot of Fig.2g. Western blotting for CFTR and β-tubulin in cells transfected with control plasmids (LacZ) or plasmids with three different CFTR-silencing sequences (CFTRkd#1,2 and 3). CFTRkd#2 was used in other experiments and is shown in Fig.2g.
